# Wearable E-Textile and CNT Sensor Wireless Measurement System for Real-Time Penile Erection Monitoring

**DOI:** 10.3390/s22010231

**Published:** 2021-12-29

**Authors:** Yongki Heo, Jinhyung Kim, Cheolung Cha, Kyusik Shin, Jihyoung Roh, Jungki Jo

**Affiliations:** 1 Department of Medical and Digital Engineering, Hanyang University, Seoul 04763, Korea; dydrlgj@hanyang.ac.kr; 2 Smart Sensor Research Center, Korea Electronics Technology Institute, Seongnam 13509, Korea; kimjh91@keti.re.kr (J.K.); cucha@keti.re.kr (C.C.); neokarion@keti.re.kr (K.S.); 3 Department of Medical Device Development Center, Daegu-Gyeongbuk Medical Innovation Foundation (DGMIF), Daegu 41061, Korea; jhr726@dgmif.re.kr

**Keywords:** erectile dysfunction, CNT, E-textile, NPT, wearable device

## Abstract

Erection measurements are the most important indicator of male urological disease diagnosis, treatment, and results. Rigiscan has been used widely in studies and diagnoses for nocturnal penile tumescence for evaluating erectile dysfunction by measuring the number and timing of erectile dysfunctions during sleep. However, this device has limitations such as the weight and bulk of the device and has been questioned for its role as a standard for ED Erectile Dysfunction (ED) diagnosis. In this study, we propose a real-time wearable monitoring system that can quantitatively measure the length and circumference of the penis using electronic textiles (E-textile) and carbon nanotube (CNT) sensors. The E-textile sensor is used to measure the length, circumference, and gradient with portability, convenience, and comfort. Sensors were created by coating CNTs on latex for flexibility. The CNT-based latex condom-type sensor in our proposed system shows the length, circumference, and curvature measurements with changes in resistance, and the E-textile performance shows a 1.44% error rate and a cavity radius of 110 to 300. The results of this conceptual study are for supplementary sensor development with a combination of new technologies with alternatives or existing methods for measuring erection function.

## 1. Introduction

As Information and Communications Technology (ICT) advances, various sizes and types of wearable devices are being developed. Wearable healthcare devices are being reinvented as new convergence innovation technologies become available in combination with sensors that have various functions and applications for mobile devices [1,2,3,4]. Medical technology continues to advance at a high rate, allowing patients to monitor their condition in real-time [5,6,7]. In particular, with the advent of wearable smart devices, mobile healthcare systems are attracting considerable attention, as they can reduce healthcare spending and improve treatment efficiency. In addition, while non-face-to-face technologies and services are expanding in various fields due to the influence of COVID-19, demand for healthcare-monitoring products to facilitate such medical care is increasing [8,9]. Despite interest in mobile medical systems and advances in new convergence technologies, methods related to erectile observation have been lacking, although this variable is a barometer of adult diseases such as high blood pressure, diabetes, and coronary artery disease [10,11,12,13,14,15,16,17]. The most important feature in diagnosing, treating, and monitoring erectile dysfunction is the accurate measurement of erectile function. Clinically used methods of measurement for erectile examinations include IIEF-5, ultrasound, the Stamp test, and Rigiscan. The International Index of Erectile Function (IIEF-5) is a representative questionnaire, but there is a high possibility of errors such as recall bias, and the Stamp test can only determine a one-time erection [18]. Rigiscan is a device widely used as a standard test method because it can measure the degree of expansion and stiffness simultaneously during sleep [19,20,21]. However, the device is heavy and bulky, limiting portability and disturbing sleep, which can affect the test results. In addition, the loop parts used for daily measurement are expensive and difficult to use at home.

In this study, we propose a wearable monitoring system suitable as a healthcare device. The device is 40 mm (w) × 30 mm (d) × 10 mm (h), can be attached to clothes in the form of a clip, and is lighter than 1 kg. More specifically, E-textile and CNT sensors are incorporated using multiple sensors of different shapes. The E-textile is easily worn, prevents contact with the human body using nonconductive fibers, ensures electrical stability, and is detachable. CNT-based condom-type sensors have flexibility, can identify the rates of change in length and thickness, and have a tensile rate of 150%. Furthermore, when at the optimal location, such a sensor measures the length, circumference, and bending information and provides real-time data through Amazon-based services (AWS)-based applications.

The remainder of this paper is organized as follows: Section 2 presents the approach used for sensor materials and methods. The results are presented in Section 3. Finally, Section 4 presents the conclusions and future work.

## 2. Materials and Methods

### 2.1. Wearable E-Textile Sensor

We aimed to measure the length and circumference of the penis before and after erection in men and constructed a condom-type sensor. The length measured when the penis is stretched in the relaxed state is almost the same as when it is erected, the average erect penis length is about 13 cm, and that of a flaccid penis is between 6.5 cm and 12 cm. The average circumference of the penis when erect was 12 cm, with about 90% of men showing 9.5 cm to 13.5 cm, and most flaccid penises were between 7.5 cm and 11 cm in circumference [22,23,24]. Conductive buttons for power transmission and sensing signal reception via an external contact point were constructed. The initial sensing unit size was 10 mm × 180 mm.

**Figure 1:**
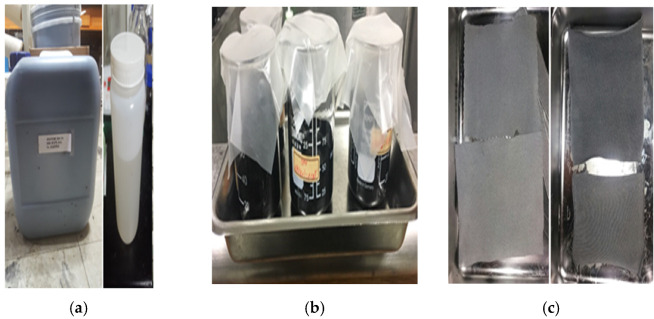
E-textile sensor fabrication process: (**a**) Carbon-based conductive particle fabrication. (**b**) Ink mixed with carbon solvents and dispersion stabilizers. (**c**) Fabrication of electrode and sensing parts.

First, improved carbon materials with less than 5% dispersion through surface treatment were developed for conductive ink manufacturing, and carbon-based conductive particles were utilized to supplement the technology of a texture-type sensor for pressure measurement, as shown in Figure 1. A highly dispersible conductive ink was mixed with carbon solvents and dispersion stabilizers to create a textile sensor that can measure pressure using a wetting process and was injected into a nonconductive textile (spandex blend) to produce a conductive textile sensor. As flexibility is required, electrode and sensing methods were developed and applied in a fabric form and sewed together. Lab-developed conductors were used to compensate for cracks that degrade electrical performance, and 100% elongated and stretchable electrode parts were developed.

A fundamental parameter of the strain gauge is its sensitivity to strain, expressed quantitatively as the gauge factor (GF). Gauge factor is defined as the ratio of fractional change in electrical resistance to fractional change in length (strain) as Equation (1):

(1) $$GF\;\left(Gauge\;Factor\right)=\frac{\frac{∆R}{R}}{\frac{∆L}{L}}=\frac{\frac{∆R}{R}}{\in }\;*\in :Materia’s\;strain$$

As the length and width of the sensor increases, the GF decreases; as the length and width decrease, it is recommended to maintain a sensor fatigue around 10 mm. In subsequent efforts, several layers or materials with a very good tensile ratio should be considered. Resistance was measured with increasing length and showed a linear increase. The resistance was measured by increasing a certain length, and it was confirmed that the resistance increased in proportion to the length change. In addition, 100% elongation is possible, so it is confirmed that the sensing unit of 10 mm × 180 mm increases to 360 mm. The electrical performance analysis of the process-optimized sensor was performed using a Universal testing machine (UTD, Dacell, Cheongju, Korea).

**Figure 2:**
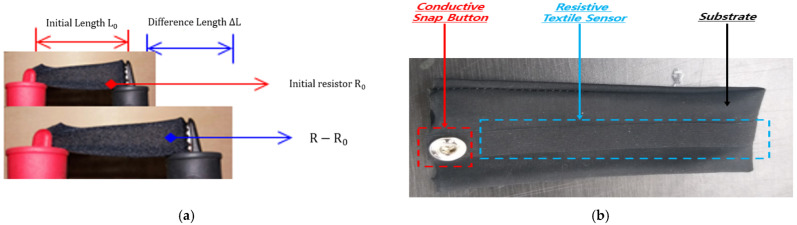
E-textile characteristics: (**a**) Gauge ratio test with gauge factors; (**b**) Resistant fiber sensor appearance.

The main consideration during development was to ensure electrical stability in a condom-shaped sensor using nonmotorized fibers as substrates to prevent direct human contact with the resistive fiber sensor, as shown in Figure 2.

### 2.2. Wearable CNT Sensor

**Figure 3:**
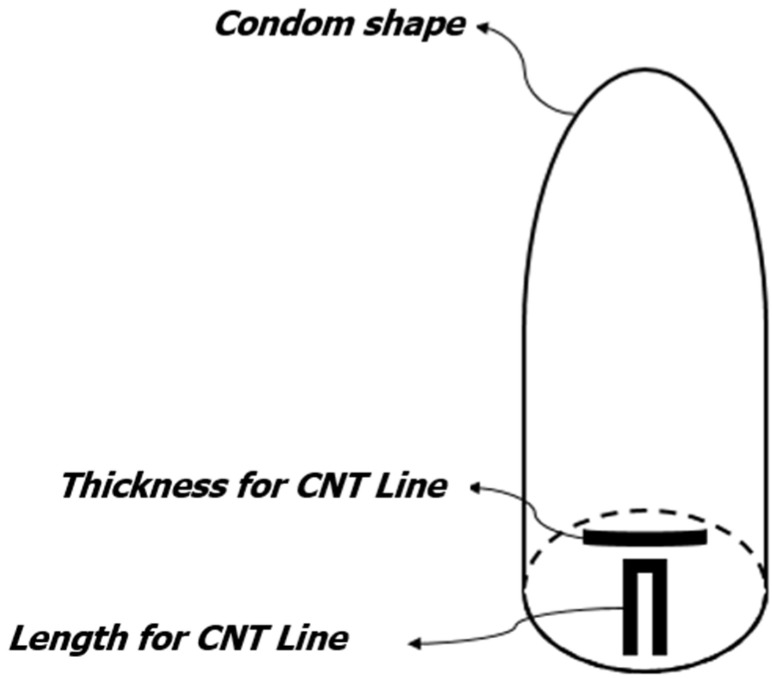
Diagram of the erection measurement sensor. CNT strip to measure length; CNT strip to measure thickness.

Figure 3 provides an overview of the proposed CNT sensor. The CNT strip for measuring penis length is placed at the bottom of the sensor, and the CNT strip for measuring thickness is placed vertically.

**Figure 4:**
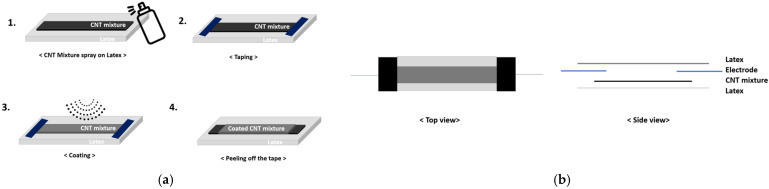
Encapsulation production process: (**a**) (**1**) CNT Mixture spray on latex. (**2**) Taping. (**3**) Latex spray coating. (**4**) Tape removal. (**b**) Erection measurement sensor structure diagram top and side views.

The sensor body comprises CNT coating on latex for flexibility and reliability. After applying a silver-paste for the initial contact point during the manufacturing process, the conductive cloth is attached with conductive thread. The initial resistance value was calculated at less than 100 kΩ for the sensor read-out IC (ROIC). The sensor can detect a length increase of 150% compared to the initial and measures the rate of change in length and thickness. At contact points between substrate and flexible sensors, cracks can occur and produce nonlinear characteristics in the resistance change graph. Contact points were manufactured, as shown in Figure 4.

**Figure 5:**
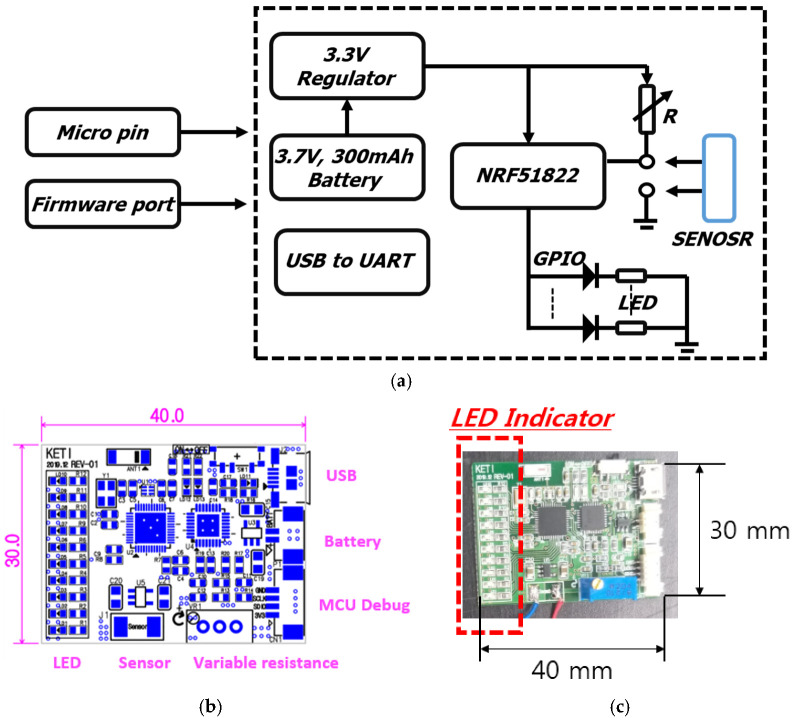
Resistant Fiber Sensor Read-out Wireless Communication Module: (**a**) Sensor module system block diagram (data transmission unit); (**b**) Communication module PCB design; (**c**) Read-out Wireless Communication Module.

In the design of the connection part, a sensor ROIC circuit was manufactured to convert the variable resistance value of the resistance sensor into a voltage value through the Voltage Divider Circuit, and a low pass filter was designed to suppress signal noise. In the control unit design, NRF51822 (Nordic)- and Coterx-M0 (MCU)-based low-power circuits were configured, and MCU- and BLE-integrated chipsets were used to minimize sensor size. An array of 10 LED indicators was used to detect changes in sensor values, as shown in Figure 5.

## 3. Results

### 3.1. Wearable E-Textile Sensor

**Figure 6:**
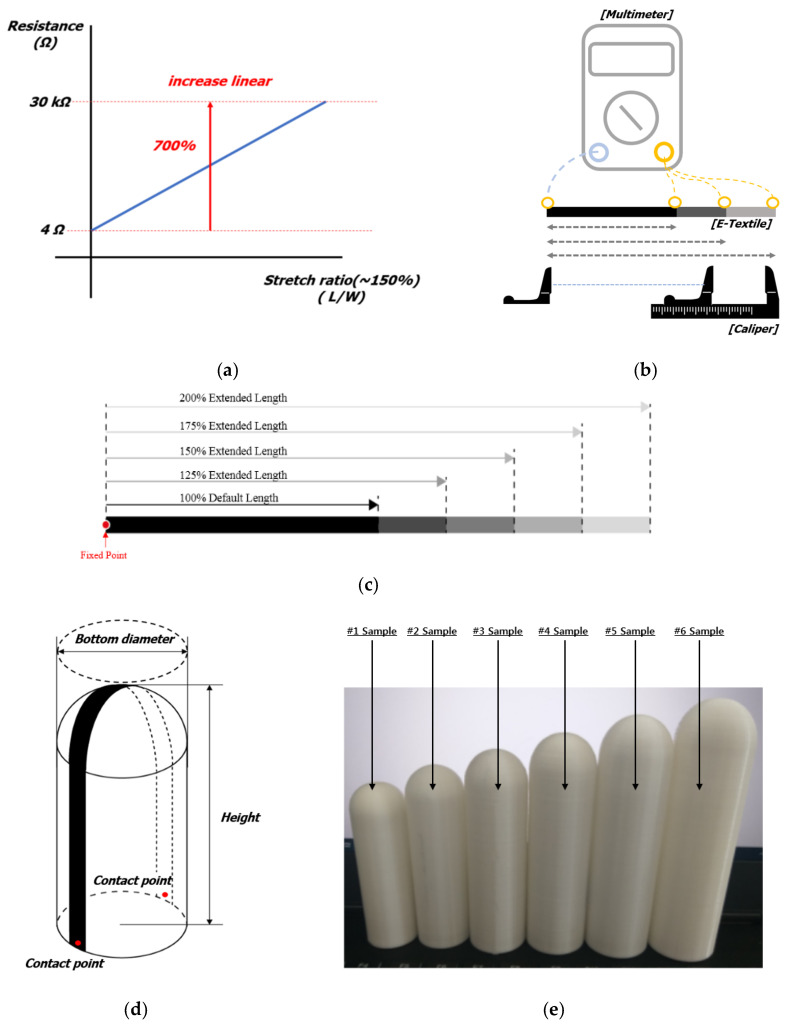
The E-textile sensor measures the resistance change according to stretch rate: (**a**) Graph of resistance change according to elongation rate; (**b**) Measurement of resistance according to stretch rate; (**c**) Change in length from 100% to 200% at a fixed point; (**d**) Mock-up sample sensor measurement schematic diagram; (**e**) Mock-up samples produced according to size.

Figure 6 shows the change in resistance according to the E-textile stretch ratio. In addition, it is possible to increase stretch by 100%, at which the sensing part of 10 mm × 180 mm extends to 360 mm. Sensor stretch was measured using multimeters and digital calipers. The modeled penis samples were manufactured using a 3D printer and a PLA filament material.

Six samples were produced based on the average erect penis size range from 28 mm to 38 mm in diameter and 90 mm to 135 mm in length, as shown in Table 1. At the contact point, one side is rigid and the other is flexible, causing cracks during stretching. These samples showed hysteresis curve-like resistance change. After raising the electrode without being fixed with a silver paste, the entire crack was covered with latex to reduce the occurrence of cracks, and a sensor with linearity was manufactured without a hysteresis curve. Latex penetrates between carbon black particles and prevents cracks from forming easily. The resistance changed as width decreased, with a 700% change compared to the initial resistance.

**Figure 7:**
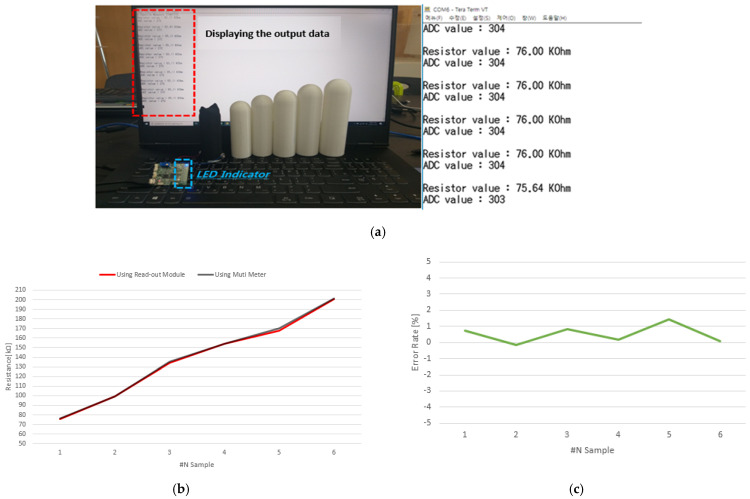
(**a**) Output data using a wireless sensor module. (**b**) Graph of comparison between measured and output resistance values. (**c**) An error graph of the output resistance value and the measured resistance value.

The sensor board outputs the analog to digital converter (ADC) value and the resistance value of the sensor through serial communication with the PC and analyzes the difference between the resistance value output of the sensor board and the resistance value measured by the Multi-Meter. The picture on the right shows the actual resistance value of the fiber sensor in the Multi-Meter. direct current (DC) and output resistance values are serials of the read-out module of the fabricated fiber sensor. The output data was recorded and the difference in the measured value–output value was expressed as a percentage (%), as shown in Figure 7.

The E-textile measurement results after using an erect penis sample are shown in Table 2. The output data of each of the six samples was compared with the measured data, and the error rate was ±0.46 %.

### 3.2. Wearable CNT Sensor

**Figure 8:**
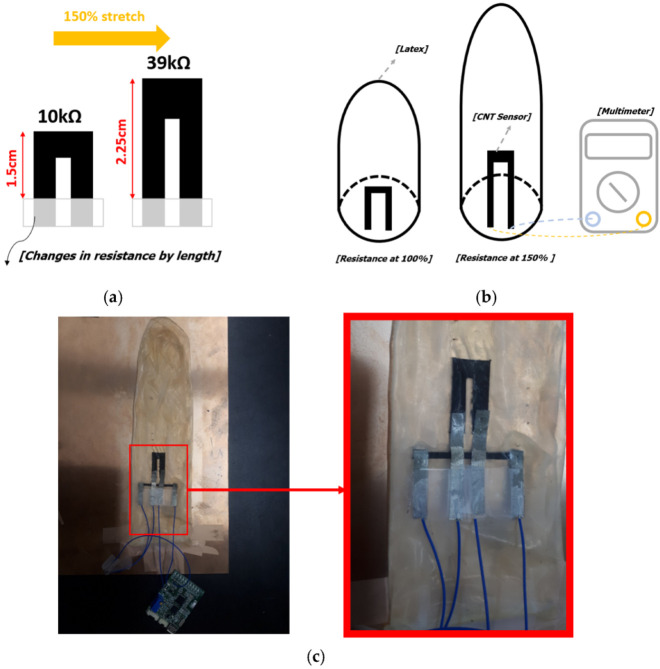
Changes in resistance according to changes in length: (**a**) Measurement of resistance change at 150% length change; (**b**) Measurement of resistance according to length of the CNT sensor; (**c**) CNT sensor-based measurement prototype configuration.

CNT-based wearable sensors measured the change in resistance value when the length was 2.25 cm, which is 150% extended based on 1.5 cm, respectively. In addition, by attaching a CNT sensor on a condom, the resistance values according to the length were measured, and, as in the previous experiments, the resistance values at 1.5 cm and 2.5 cm were measured, and the values of the multimeter and the manufactured controller were compared, respectively. Through the two comparative experiments, it was confirmed that the resistance value changed from 10 kΩ to 39 kΩ, as in the previous experiment. In order to measure the length and circumference, the condom was shaped in a ‘C’ shape in the transverse and longitudinal directions of the condom, which is for contact points, as shown in Figure 8.

When bent to the left, 10% changes were observed from 100% to 150% in the samples, and the overall resistance value of the sensor was determined as the ratio of the resistance on the left and right sides, according to the ratio of curvature.

**Figure 9:**
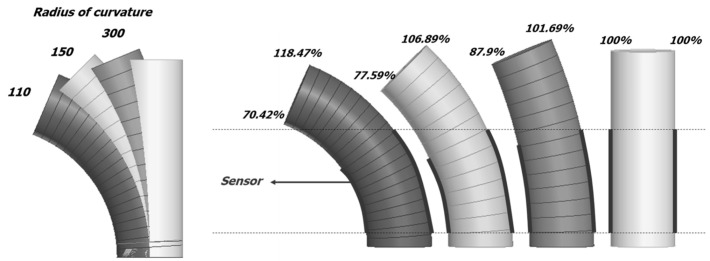
The rate of change in the lengths for the inside and outside surfaces according to change in curvature.

As shown in Figure 9, the changes in elongation and resistance were measured at a radius of curvature of 110, 150, and 300. A sensor was placed at each side of the sample and was measured, as shown in Figure 7. The inside length in the bending direction was shortened and maintained the initial resistance, while the outside length tended to increase, as shown in Table 3.

## 4. Discussion

The increase in accessibility of wearable consumer devices that generate health information provides an unprecedented opportunity to revolutionize healthcare for researchers, clinicians, and consumers [25,26,27]. In this study, to propose a new penis measurement system, we measured the length and circumference across a wearable sensor.

This is the first study to measure the length and circumference of wearable sensor forms in combination. Furthermore, our device allows the measurement of the length, thickness, and slope compared to previous efforts made at measuring the change in length of the penis. Currently, there is no standardized male penis measurement method, and there is no reliable study to measure changes before and after erection. This is relevant for clinicians who prescribe medical treatment [28,29,30] and particularly appropriate for clinicians conducting treatments or studies based on patient data. Accordingly, the clinician can obtain reliable measured data instead of a subjective questionnaire completed by the patient. Wearable technology is expected to innovate medical services. As a super-aged society, male menopause is an important issue to evaluate, and the measurement of erectile function is key to this end [31]. Through the development of a wearable erectile function scanning system, it is possible to quantitatively measure the angle before and after treatment in Peyronie’s disease [32,33], which had been measured previously using a protractor [34]. In addition, the number of patients with urinary and chronic diseases is increasing, and medical access opportunities can be provided to patients where visitation is not possible due to social or psychological difficulties. Erection occurs after prostatectomy, and it is necessary to measure it quantitatively. Furthermore, restoring erectile dysfunction through drug administration such as Viagra is called penile rehabilitation, which means quantitatively measuring its effectiveness. In addition, chronic diseases refer to cardiovascular disease or cerebrovascular disease. Moreover, social costs are reduced due to early diagnosis, and increased service can offer medical care to vulnerable areas. Studies are measuring the length, thickness, and angle with which to reliably measure changes in the penis while minimizing user inconvenience and heterogeneity during use.

We expect this study to help researchers and clinicians and improve health through the accurate health monitoring of patients and the general public.

## 5. Conclusions

This study presents a quantitative penis measuring device that utilizes an E-textile and a CNT sensor, as well as a mobile healthcare system that enables real-time monitoring from smart devices. In addition, sleep disturbance has been minimized by the improved miniaturization and wearability of the device. Moreover, the device is simple enough to be used at home without the aid of a doctor, and the data can be saved and accessed in real-time, which allows remote healthcare services. Furthermore, we expect to create more reliable sensors and optimize signal processing algorithms. These advances are expected to be applied to chronic diseases such as male rehabilitation monitoring or cardiovascular diseases.

With our system, revolutionary improvements are expected in measurement tools for erectile dysfunction and health problems related to erection measurements.

## Figures and Tables

**Table 1:** Mockup sample measurements.

	Sample #1	Sample #2	Sample #3	Sample #4	Sample #5	Sample #6
Diameter(mm)	28	30	32	34	36	38
Height(mm)	90	99	108	117	126	135

**Table 2:** The output data of each of the six samples.

	Sample #1	Sample #2	Sample #3	Sample #4	Sample #5	Sample #6
Output resistance (kΩ)	75	99	134	153	169	200
Measured resistance (kΩ)	76	99	135	154	170	201
Error (%)	0.73	−0.17	0.82	0.19	1.44	0.08

**Table 3:** Resistance changes according to changes in length of inside and outside sensors.

	300 (Left/Right)	150 (Left/Right)	110 (Left/Right)
100%	17.5 kΩ/45.6 kΩ	17.58 kΩ/45.6 kΩ	17.5 kΩ/45.6 kΩ
110%	19.3 kΩ/186.5 kΩ	17.07 kΩ/231.1 kΩ	15.4 kΩ/280.6 kΩ
120%	103.3 kΩ/256.0 kΩ	18.62 kΩ/274.8 kΩ	16.9 kΩ/312.9 kΩ
130%	205.3 kΩ/284.3 kΩ	20 kΩ/297.9 kΩ	18.31 kΩ/380 kΩ
140%	259.8 kΩ/314.2 kΩ	151.1 kΩ/325.5 kΩ	19.72 kΩ/455.1 kΩ
150%	255.6 kΩ/375.2 kΩ	221.8 kΩ/422 kΩ	105.5 kΩ/526.2 kΩ

## Data Availability

Not applicable.
